# Characterization of the complete chloroplast genome of the *Pistacia vera* L

**DOI:** 10.1080/23802359.2019.1692723

**Published:** 2019-11-21

**Authors:** Weitao Mao, Guoxin Yao, Bo Chen, Qinghua Pan, Hai Wang, Guanglong Hu, Guo Xin

**Affiliations:** a Hubei Key Laboratory of Quality Control of Characteristic Fruits and Vegetables, College of Life Science and Technology, Hubei Engineering University, Xiaogan, China;; b School of Life Science, Hubei University, Wuhan, China;; c Beijing Academy of Forestry and Pomology Sciences, Beijing, China;; d Walnut Research Institute, Longnan Economic Forest Research Institute, Longnan, China

**Keywords:** *Pistacia vera* L, chloroplast genome, Illumina sequencing, phylogenetic analysis

## Abstract

The complete chloroplast genome sequence of *Pistacia vera* was mapped and determined based on Illumina sequencing data. The complete chloroplast genome is 160,654 bp and contains a pair of inverted repeat regions of 26,596 bp each, a large single-copy region of 88,376 bp, and a small single-copy region of 19,086 bp. It harbors 113 genes, including 79 protein-coding genes,4 ribosomal RNA genes, and 30 transfer RNA genes. Phylogenetic analysis based on chloroplast genomes indicates that *Pistacia vera* is closely related to that of *Pistacia weinmanniifolia.*

Pistachio *(Pistacia vera* L.) belongs to the genus Pistacia and the family Anacardiaceae, which is one of the most important nut crops and cultivated widely in the Mediterranean regions of Europe, North Africa, Middle East, west of China, and USA (Zohary 1952; Sheibani 1995; Onay et al. 2004). Although several chloroplast (cp) DNA markers have previously been used for the phylogenetic analysis of *P. vera* (Sarra et al. 2015; Talebi et al. 2016), little is known about the cp genome of *P. vera*, and there are different views on the taxonomy of this species (Al-Saghir 2009). In the present study, we report the first complete cp genome sequence of *P. vera* based on Illumina paired-end sequencing data (GenBank accession number MN551174).

Fresh leaves were collected from a single *P. vera* tree (*P. vera* var Kerman) growing in Huangjiaba (104°51′E, 33°25′N), Chengguan town, Wudu District, Longnan City, Gansu province, China. Voucher specimens were deposited at the herbarium of the Longnan Economic Forest Research Institute (accession number: 20190701GL). DNA extraction was performed according to a modified CTAB protocol (Li et al. 2013). High-throughput sequencing was carried out using the HiSeq2500 PE150 System (Illumina, San Diego, CA, USA). The cp genome assembly with SPAdes 3.6.1 (Bankevich et al. 2012) and Sequencher 4.10 (https://www.genecodes.com/) software tools. Reference-guided assembly was then performed to reconstruct the cp genome with the BLAST program (Altschul et al. 1990) using closely related species as references. After filling the gaps with GapCloser (http://soap.genomics.org.cn/), a 160,654 bp cp genome was obtained for *P. vera.* Annotation was performed using the Plann (Huang and Cronk 2015), then a physical map of the cp genome generated by Genome Vx (Conant and Wolfe 2008).

The circular cp genome of *P. vera* contains a pair of inverted repeat(IR) regions of 26,596 bp each, and large single-copy (LSC) and small single-copy (SSC) regions of 88,376 bp and 19,086 bp, respectively. The genome comprises 113 genes, including 79 protein-coding, 30 transfer RNA genes, 4 ribosomal RNA genes (16S, 23S, 5S, 4.5S). Among the annotated genes,19 genes contained introns, including 17 with a single intron each and two with two introns each (*clpP* and *ycf3*). There is a trans-splicing gene (rps12) whose 5′ exon is located in the LSC and 3′ exon is located in the IR region.

To identify the phylogenetic position of *P. vera*, a maximum likelihood analysis was performed based on 80 coding genes in the chloroplast genome of 10 samples of Anacardiaceae and 22 other related plants using IQ-TREE software (Nguyen et al. 2015). The cp genome of *P. vera* was shown to be similar to that of *Pistacia weinmanniifolia* and closely related to that of *Rhus chinensis* of the family Anacardiaceae (Figure 1). This complete cp genome can be used for subsequent population and cp genetic engineering studies, and especially to determine the phylogenetic position of *P. vera* in *Pistacia* L.

**Figure 1.**
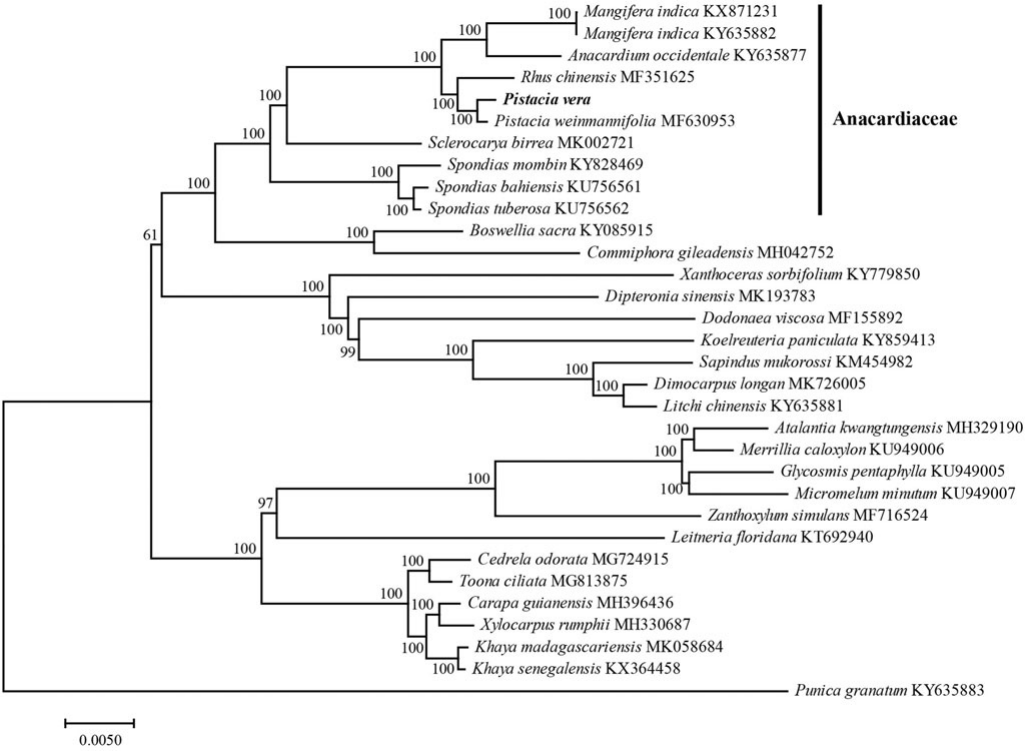
Phylogenetic tree inferred using the IQ-TREE software based on 80 coding genes from 32 complete chloroplast genomes.
